# Two novel porcine teschovirus strains as the causative agents of encephalomyelitis in the Netherlands

**DOI:** 10.1186/s12917-020-2275-0

**Published:** 2020-02-11

**Authors:** Sandra Vreman, Nermin Caliskan, Frank Harders, Jan Boonstra, Klaas Peperkamp, Cynthia K. Y. Ho, Wikke Kuller, Jeroen Kortekaas

**Affiliations:** 1 Wageningen Bioveterinary Research, Wageningen University & Research, P.O. Box 65, 8200 AB Lelystad, The Netherlands; 2 Department of Pathobiology, Utrecht University, Utrecht, the Netherlands; 3 GD Animal Health, Deventer, the Netherlands; 4 University Farm Animal Practice (ULP), Harmelen, the Netherlands; 5 Laboratory of Virology, Wageningen University, Wageningen, the Netherlands

**Keywords:** Porcine teschovirus, Weanling pigs, Non-suppurative encephalomyelitis

## Abstract

**Background:**

Porcine teschovirus (PTV) circulates among wild and domesticated pig populations without causing clinical disease, however neuroinvasive strains have caused high morbidity and mortality in the past. In recent years, several reports appeared with viral agents as a cause for neurologic signs in weanling and growing pigs among which PTV and new strains of PTV were described.

**Case presentation:**

On two unrelated pig farms in the Netherlands the weanling pig population showed a staggering gate, which developed progressively to paresis or paralysis of the hind legs with a morbidity up to 5%. After necropsy we diagnosed a non-suppurative encephalomyelitis on both farms, which was most consistent with a viral infection. PTV was detected within the central nervous system by qPCR. From both farms PTV full-length genomes were sequenced, which clustered closely with PTV-3 (98%) or PTV-11 (85%). Other common swine viruses were excluded by qPCR and sequencing of the virus.

**Conclusion:**

Our results show that new neuroinvasive PTV strains still emerge in pigs in the Netherlands. Further research is needed to investigate the impact of PTV and other viral agents causing encephalomyelitis within wild and domestic pig populations supported by the awareness of veterinarians.

## Background

The family *Picornaviridae* (order *Picornavirales*) comprises 110 named positive-strand RNA viruses, grouped into 47 genera [1]. In addition to enteroviruses, sapeloviruses and teschoviruses, many picornaviruses have been detected in pigs: Foot-and-mouth disease viruses, encephalomyocarditis viruses, porcine cosaviruses, kobuviruses, pasiviruses, senecaviruses and tottoriviruses. The genus *Teschovirus* has been frequently associated with neurologic disease in pigs [2–5]. Porcine teschovirus (PTV) currently comprises 13 known serotypes [6–8], which is likely to expand with the continuous discovery of novel genotypes [9, 10].

PTV is globally endemic in pig herds and is commonly isolated from fecal samples of clinically healthy wild and domestic pigs [11, 12], indicating that these viruses replicate in the intestinal tract without causing disease. However, when PTV invades the central nervous system (CNS) it can cause severe neurologic signs [13]. The first outbreak of severe CNS disease associated with PTV serotype 1 (PTV-1) occurred in 1929 in the Czech Republic in a town named Teschen [13, 14]. In 1957, a PTV-1 strain of lower pathogenicity caused an outbreak near the Talfan hill in Wales [15, 16]. Since that time, severe PTV-1-associated CNS disease is referred to as Teschen disease and the milder clinical form as Talfan disease. The most recent severe outbreak of PTV-1 with high mortality occurred in the Republic of Haiti in 2009 [17]. However, this virulent manifestation is now considered rare and only milder forms of neurologic disease associated with PTV-1 or other PTV serotypes have been reported in growing pigs in Canada, Spain and Brazil during the last years [2, 5, 8]. Although co-infections were not detected in these cases, immunosuppressive pathogens, such as porcine circovirus type 2 (PCV-2) and/or porcine reproductive and respiratory syndrome virus (PRRSV) could predispose animals to develop neurologic PTV disease [3, 18]. It is therefore important to screen for immunosuppressive pathogens when PTV is suspected to be associated with neurologic signs.

## Case presentation

In 2014 and 2015, weanling piglets between the age of 6–12 weeks from two commercial pig farms showed periodically neurological signs. On both farms, the affected weanling piglets had a staggering gait, which developed progressively in 1 or 2 days from paresis to complete paralysis of the hind limbs. Paralyzed animals died within a few days or were euthanized. On farm A, a farrow-to-finish farm with more than 4000 animals (Topigs20), these neurologic signs occurred over a period of more than 5 years, during which the morbidity of the weanling piglets varied from 0 to 5%. On this farm no other significant health issues were noticed. On farm B, a fattening farm with 2000 animals (Topigs 40 x Pietrain), similar neurologic signs were noticed in pigs obtained repeatedly from one specific farrow farm. No further information is available for farm B. Both farms belong to separate companies and are located in two different parts of the Netherlands (> 100 km distance).

Clinical differential diagnoses for these neurologic signs include infectious (viral and bacterial) and non-infectious causes, such as tail-biting, sodium chloride intoxication and poisoning. Tail-biting was excluded based on clinical examination, and water nipples and water quality were checked regularly to prevent water deprivation, causing sodium chloride intoxication. No medication was used nor any change in feed was made, which makes poisoning unlikely. Only on farm A, the animals with comparable neurologic signs were tested repeatedly negative for *Streptococcus suis* and *Escheria coli*, which were considered the most likeley differential diagnosis. On the other hand, as ascending paresis/paralysis was the most typical clinical sign in the affected piglets bacterial agents causing septicaemia, like *Streptococcus suis, Haemphilus parasuis* or edema disease-associated *Escheria coli*, were considered unlikely. Also possible viral causes, like Suid herpesvirus type 1 (SuHV-1) and classical swine fever (CSF), were less likely based on clinical signs and/or the fact that clinical signs were observed over a long period of time.

**Fig. 1:**
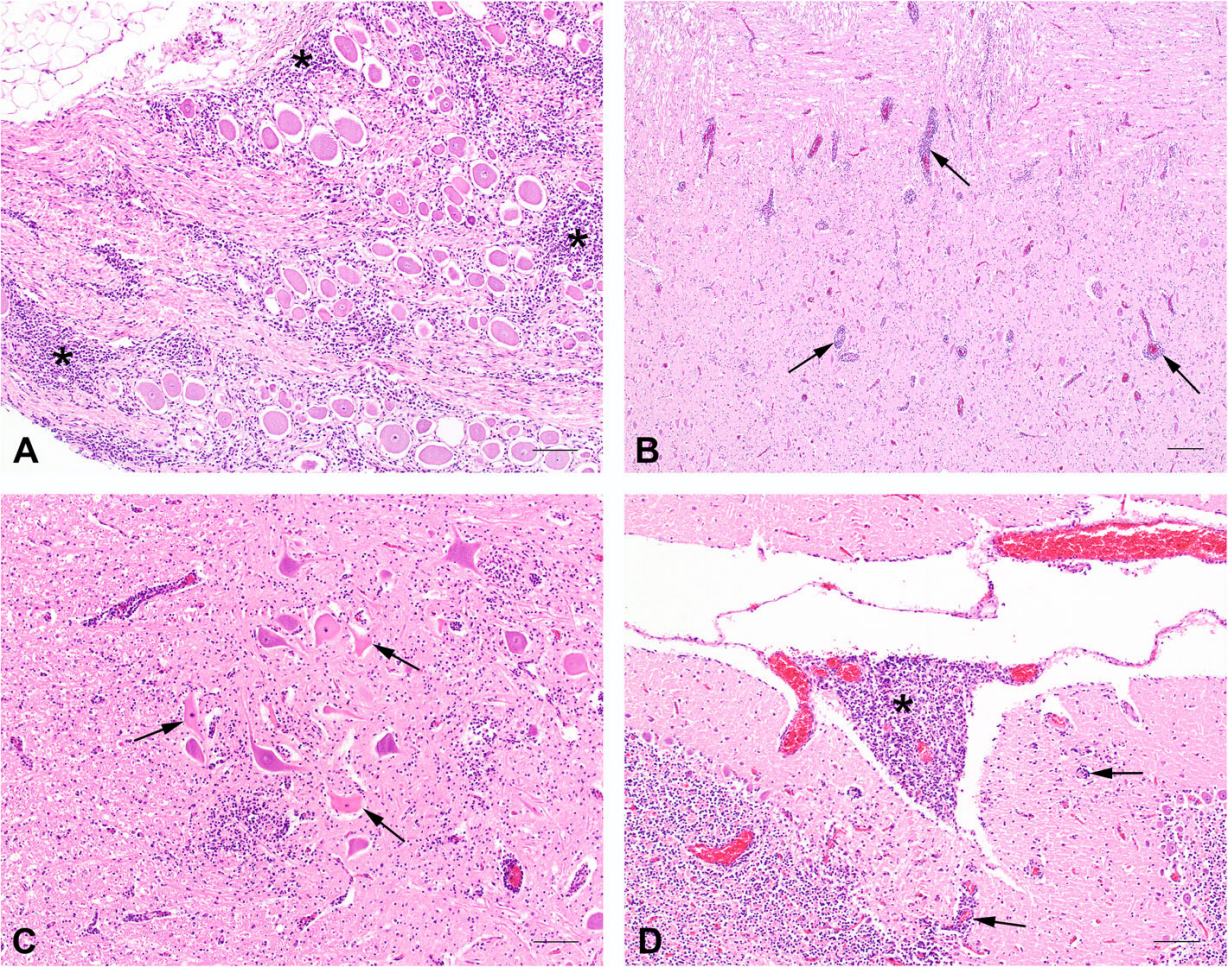
Sections of the central nervous system and trigeminal ganglion. Tissue sections from one animal from farm A stained with hematoxylin and eosin (H&E). **a** Trigeminal ganglion with infiltration of a large number of macrophages and lymphocytes (asterisk) (Bar = 100 μm); (**b**) Proximal spinal cord (gray matter) with perivascular infiltrates (perivascular cuffing; arrow) of mainly macrophages, lymphocytes and a lesser number of plasma cells, extending into the neuropil (Bar = 200 μm); (**c**) Cerebrum (gray matter) with perivascular cuffing, gliosis and neuronal degeneration and necrosis (arrow) (Bar = 100 μm); (**d**) Cerebellum with infiltration of a large number of lymphocytes and macrophages into the meninges (asterisk), and perivascular cuffing in the parenchyma (arrow) (Bar = 200 μm)

Piglets were submitted for pathological examination to identify the underlying cause of the neurologic signs. In total, six weanling piglets with clear clinical signs, aged between 7 and 10 weeks, from the two different farms (four animals from farm A and two animals from farm B) were necropsied. None of these piglets showed macroscopic changes. Tissue samples from lung, heart, kidney, liver, gastro-intestinal tract, trigeminal ganglion (only farm A) and CNS (spinal cord, cerebellum and cerebrum) were formalin-fixed and paraffin-embedded and tissue sections were stained with hematoxylin and eosin (H&E). In all animals histological changes were confined to the CNS and trigeminal ganglia. The trigeminal ganglion of the animals (farm A) showed infiltrates of mainly lymphocytes and macrophages around the neurons (Fig. 1a). The spinal cord, cerebrum and cerebellum showed perivascular infiltrates up-to 7 layers composed primarily of macrophages, lymphocytes and fewer plasma cells (mononuclear infiltrate Fig. 1b, c and d) and occasional eosinophils. These infiltrates extended into the neuropil where gliosis with glia nodules, neuronal degeneration and necrosis were encountered. The CNS lesions were most prominent in the gray matter of the spinal cord, where the lesions were bilateral and asymmetric. The cerebrum and cerebellum showed less severe changes (Additional file 1: Table S1). The meninges were mildly infiltrated with mainly macrophages and lymphocytes, which was more prominent around the brain compared to the spinal cord (Fig. 1d). Although there was variation in severity between the individual animals, there was no significant difference in morphology or severity of the CNS histopathology in the pigs of both farms. The observed histological changes were highly suggestive of a viral infection in the CNS. Possible viral causes of this non-suppurative or lymphohystiocytic encephalomyelitis in Western-European pigs without any other significant pathology could include: SuHV-1 (also known as Aujeszky’s disease or pseudorabies), PTV, PCV-2 and PRRSV [19, 20], and more recently described porcine sapelovirus (PSV) [21–23] and porcine astrovirus (PoAstV) [24, 25]. No additional research was performed to exclude bacterial pathogens, as a bacterial cause was highly unlikely based absence of gross inflammatory changes and the morphology the histopathological findings.

To identify a potential viral cause, brain and spinal cord tissues were investigated further. First an immunoperoxidase monolayer assay (IPMA) was performed on a monolayer of porcine kidney epithelial (PK15) cells incubated with 10% spinal cord or brain suspensions using serotype-specific hyperimmune sera. As PTV-1 is a notifiable disease in the Netherlands, the IPMA was performed with an antiserum specific for this serotype. As a negative control, a serum specific for PTV-2 was included. The IPMA was negative for PTV-1 and PTV-2, although a clear cytopathogenic effect (CPE) was observed in the cell cultures, suggesting the presence of a cytopathogenic agent.

Consecutively, we performed a real-time qPCR for PTV, PCV-2, PRRSV and suHV-1with hybridization probes specific for the amplification products. DNA or RNA was isolated from brain and spinal cord tissues by column chromatography. The primers and probe for PTV were specific for the 5′ untranslated region (5‘UTR), which is conserved among different serotypes of PTV [26], and were used with slight modifications in the primers and probes. The other viral qPCRs were used as described by van Rijn et al., 2004 [27] and Wellenberg et al., 2004 [28]. Primer and probe sequences are provided as Additional file 1: Table S2. Positive controls derived from cell-grown stocks of the different viral agents were included. All tested brain and/or spinal cord samples from farm A and B were positive for PTV (Cq values between 25.3–34.0 for the different CNS samples (Additional file 1: Table S3); Cq value of positive control for PTV is 29.0, low dilution control, and 35.5, high dilution control), whereas none of the other viral agents (PRRSV, PCV2, suHV-1) were detected.

**Fig. 2:**
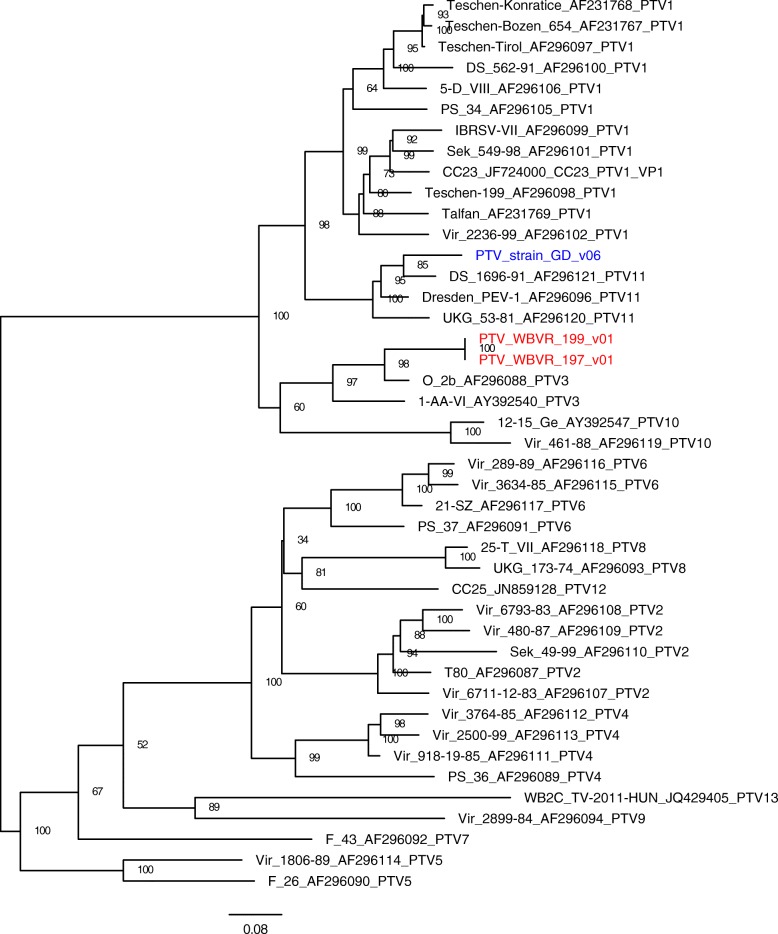
The maximum likelihood (ML) phylogeny of Porcine teschovirus (PTV) VP1. The gene of the three isolates (red and blue) and 40 references sequences with bootstrap values. The 40 reference sequences are labeled by name, GenBank accession number and serotype. The three PTV strains of interest are colored blue (PTV_strain_GD_v06) and red (PTV_WBVR_197_v01 and 199)

Supernatants of cell lysates from the cultures in which CPE was detected were used to determine full genome sequences of the PTV isolates. Near full-length sequences of the three isolates were generated using the Illumina sequencing platform. A phylogenetic tree of the VP1 gene was reconstructed (Fig. 2) using the maximum likelihood method with 1000 bootstrap replicates and substitution model TIM2 + F + I + G4 in IQ-TREE v1.6.8 [29]. To determine similarity with known PTV serotypes, 40 reference PTV sequences of serotypes 1–13 were obtained from GenBank and included in the analyses. The phylogenetic tree was annotated in FigTree v1.4.3 http://tree.bio.ed.ac.uk/software/figtree/. From farm A, the PTV_WBVR_197_v01 and 199 strains from two different animals (identical in nucleotide sequence) clustered closely with the PTV-3 serotype with a high support value (98%) and PTV_strain_GD_v06 from farm B clustered closely with serotype 11 with a support value of 85%. The genome sequences are available on the European Nucleotide Archive (ENA) (PTV_197 as ERS3526779; PTV_199 as ERS3526780 and PTV_GDv06 as ERS3526781).

The alignment of the sequence data against the National Center for Biotechnology Information (NCBI) viral database followed by a manual inspection with the Integrative Genomics Viewer version 2.5.2 excluded the presence of PSV and PoAstV in the sequence data of both farms.

## Discussion and conclusion

On two farms in the Netherlands with neurologic signs in weanling piglets we identified PTV as causative agent. This diagnosis was based on the location (prominent lesions in spinal cord) and the morphology of CNS histopathology and concurrent positive virus isolation and identification. Additional differential viral infections were excluded by absence of histopathological lesions in lung and lymph nodes, qPCR (PRRSV, PCV and suHV-1) and virus sequencing (PSV and PoAstV). Based on phylogenetic analyses we concluded that our isolated PTV strains are closely related to serotype 3 (strains WBVR_197_v01 and 199), first described in the USA (Ohio) [30] and serotype 11 (strain_GD_v06), first described in Europe (Dresden, Germany) [6]. Of note, PTV-3 [31] and PTV-11 [32] were previously shown to be neuropathogenic after experimental inoculation, manifesting with comparable clinical signs and histopathological changes in the CNS. Similar as described in this case report, the histopathological changes were most prominent in the spinal cord and less severe in cerebellum and cerebrum. Based on distribution, severity or morphology of the CNS histopathology we were unable to discriminate between the two newly identified PTV strains.

The *Picornaviridae* family contains small RNA viruses and their pathogenicity is associated with their ability to undergo mutation and genetic recombination, causing a high diversity over a short period of time [33]. Recombination of PTV strains have been reported [34], which may contribute significantly to the evolution of PTV. It is not known whether our isolated strains have evolved towards higher virulence or that their pathogenic potential was not recognized until today.

In several countries worldwide, such as Spain and Brazil, an increased prevalence of PTV-associated neurological disease is noted [2, 8]. Recent reports have identified other viral causes of encephalomyelitis in pigs of similar age. Eleven-week-old North American piglets infected with PSV presented comparable neurological signs as we have observed in the present work, and were diagnosed with lymphoplasmacellular and necrotizing polioencephalomyelitis [21, 22]. In 2017, PSV was detected during several porcine epidemic diarrhea outbreaks in Germany, noting the possible presence in the European commercially held swine population [35]. In Hungary, PoAstV type 3 was found in piglets with posterior paraplegia, encephalomyelitis and high mortality rates, although the authors were cautious in making a direct association between the virus and the central nervous lesions [24]. To the author’s knowledge, no research on PSV and PoAstV infections has been performed in swine herds in the Netherlands and in our report these viral causes were highly unlikely based on sequencing analyses.

Despite the fact that the encephalomyelitis was suggestive for a viral infection and that PTV was the only identified pathogen in the affected pigs, we have to recognize that not all Koch’s postulates were met in this case report, because this were all diagnostic cases. Investigations of future encephalomyelitis outbreaks would be facilitated greatly by including healthy age-matched control pigs. To our knowledge, no new cases of PTV have been diagnosed in the Netherlands since 2015. As only PTV-1 is a notifiable virus in the Netherlands, different strains of PTV could remain underreported or underdiagnosed. However, despite the low mortality on the farms investigated in the present work, it is very important that veterinarians are aware of this disease as the viruses may have the potential to evolve into more pathogenic strains. The objective of this report is to describe the diagnostic process for neuropathogenic PTV, where histopathology and additional molecular diagnostics are essential, and raise awareness for PTV and other viral agents, such as PSV and PoAstV causing encephalomyelitis in wild and domestic pig populations. Further research on pathology and epidemiology would facilicate assessment of the impact of PTV on global pig health and the swine industry.

In summary, on two different farms in the Netherlands, two new PTV strains were identified within the CNS of weanling piglets after earlier histopathological diagnosis of a lymphohistiocytic encephalomyelitis. Further research is needed to investigate the incidence and relevance of PTV and other viral agents, such as PSV and PoAstV causing encephalomyelitis within wild and domestic pig populations supported by the awareness of veterinarians and research institutes.

## Supplementary information

**Additional file 1:Table S1.** Overview histopathology central nervous system for animals farm A and B. **Table S2.** Primer and probe overview for qPCR. **Table S3.** Overview animals and analysis.

## Data Availability

The viral sequences are deposited in ENA (PTV_197 as ERS3526779; PTV_199 as ERS3526780 and PTV_GDv06 as ERS3526781). Phylogeny data, including alignments, are deposited in the TreeBASE repository http://purl.org/phylo/treebase/phylows/study/TB2:S25306. Other datasets used in the current study are available from the corresponding author on reasonable request.

## Abbreviations

CNS: Central nervous system
CSF: Classical swine fever
ENA: European Nucleotide Archive
IPMA: Immunoperoxidase monolayer assay
NCBI: National Center for Biotechnology information
PCV-2: Porcine circovirus type 2
PoAstV: Porcine astrovirus
PRRSV: Porcine reproductive and respiratory virus
PSV: Porcine sapelovirus
PTV: Porcine teschovirus
qPCR: Quantitative polymerase chain reaction
SuHV-1: Suid herpesvirus type 1
