# Supplementary material for: Two novel porcine teschovirus strains as the causative agents of encephalomyelitis in the Netherlands
Source: BMC Vet Res. 2020 Feb 11;16:51. doi: 10.1186/s12917-020-2275-0 (PMC7014746; doi:10.1186/s12917-020-2275-0)
Supplement: Supplementary file 1 — Additional file 1:Table S1. Overview histopathology central nervous system for animals farm A and B. Table S2. Primer and probe overview for qPCR. Table S3. Overview animals and analysis. [file 12917_2020_2275_MOESM1_ESM.docx]

**Table S1: Overview histopathology central nervous system for animals farm A and B**

|  |  | Spinal cord | Cerebellum | Cerebrum | Trigeminal ganglion |
| --- | --- | --- | --- | --- | --- |
| Farm A | Pig 1 | **+++** | **++** | **++** | **++** |
| Farm A | Pig 2 | **+++** | **++** | **++** | **++** |
| Farm A | Pig 3 | **+** | **+** | **0** | **0** |
| Farm A | Pig 4 | **++** | **++** | **+** | **++** |
| Farm B | Pig 1 | **+++** | **++** | **+** | **np** |
| Farm B | Pig 2 | **+++** | **++** | **+** | **np** |

**Table S1: Overview histopathology central nervous system for animals farm A and B.** The severity of the histopathological changes (absent 0, mild +, moderate ++ or severe +++) in different parts of the CNS (spinal cord, cerebellum and cerebrum) and the trigeminal ganglion was evaluated for the different animals from farm A and farm B; np= not present.

**Table S2: Primer and probe overview for qPCR**

| Virus | Sequence (5’ 3’) | Genome region | reference |
| --- | --- | --- | --- |
| PTV | F: GGC-GAC-AGG-GTA-CAG-AAG-AG  R: CTGT CAG GCA GCA CAA GT  Probe: 6FAM-AGATTTCTCTGGGGCCCACC-TMR | 5’UTR | Jimenez-Clavero et al. 2003 [26] |
| PRRSV | F: ATG-KGG-CTT-CTC-MGG-STT-T  R: ATG-GCC-AGC-CAG-TCA-A-X-TC-3  Probe: GCA-CCC-AGC-AAC-TGG-CAC-AGT-TGA-fl- | Junction ORF6 and ORF7 | van Rijn et al., 2004 [27] |
| PCV-2 | F: GGG-CCA-GAA-TTC-AAC-CTT-AAC-CT  R: CTC-TCC-CGC-ACC-TTC-GGA-X-TAT  Probe : CGT-TGT-GAC-TGT-GGT-WSS-CTT-GAY-AGT-fl | Capsid protein | Wellenberg et al., 2004 [28] |
| SuHV-1 | F: GCT TCC ACT CGC AGC TC  R: GTA GAT GCA GGG CTC GTA CA  Probe: 6FAM-CCGGGGACACGTTCGACCTG-TMR | Glycoproteine E | van Rijn et al., 2004 [27] |

**Table S2: Primer and probe overview for qPCR.** real-time qPCR with hybridization probes specific for the amplification products. Abbreviations virus: porcine teschovirus (PTV), porcine reproductive and respiratory syndrome virus (PRRSV), porcine circovirus type-2 (PCV-2) and Suid herpesvirus type 1 (SuHV-1).

**Table S3: overview animals and analysis.**

|  | Farm A | Farm B |
| --- | --- | --- |
| Institute | Utrecht University/WBVR | GD Deventer |
| Animals | n=4 | n=2 |
| Pathology | Positive (n=4) | Positive (n=2) |
| IPMA PTV-1 and PTV-2 | Negative (n=4) | Negative (n=2) |
| qPCR PTV (Cq value)  Spinal cord/ brain | Pig 1 – 25/32  Pig 2 – 30/31  Pig 3 – 28/33  Pig 4 – not tested/32 | Pig 1 -27/not tested  Pig 2 – not tested |
| qPCR PRRSV, PCV-2 and SuHV-1 | Not detected (n=4) | Not detected (n=2) |
| NGS-PTV-strain | Pig 2 – PTV_WBVR_197_v01  Pig 3 - PTV_WBVR_199_v01 | Pig 1 – PTV_GD_v06 |
| Alignment of NSG data for PSV and PoAstV | Pig 2 and 3  Not detected | Pig 1  Not detected |

**Table S3: Overview animals and analysis.** Institutes where necropsies and histopathology were performed: Utrecht University, Faculty of Veterinary Medicine, department pathobiology; Wageningen Bioveterinary research, Wageningen University and Research and GD Deventer). The immunoperoxidase monolayer assay (IPMA), qPCRs and next generation sequencing (NSG) were performed at WBVR (national reference laboratory for porcine teschovirus (PTV-1); abbreviations: porcine reproductive and respiratory syndrome virus (PRRSV), porcine circovirus type-2 (PCV-2), Suid herpesvirus type 1 (SuHV-1), porcine sapelovirus (PSV) and porcine astrovirus (PoAstV).
